# 'Candidatus Phytoplasma vignae’, assigning a species description to a long-known phytoplasma occurring in northern Australia

**DOI:** 10.1099/ijsem.0.006502

**Published:** 2024-08-27

**Authors:** Bianca Rodrigues Jardim, Lucy T. T. Tran-Nguyen, Cherie Gambley, Craig Webster, Monica Kehoe, Samantha Bond, Brendan Rodoni, Fiona E. Constable

**Affiliations:** 1 School of Applied Systems Biology, La Trobe University, Bundoora, Victoria, Australia; 2 Agriculture Victoria Research, Department of Energy, Environment and Climate Action, AgriBio, Bundoora, Australia; 3 Plant Health Australia, Deakin, Australian Capital Territory, Australia; 4 Horticulture and Forestry Science, Department of Agriculture and Fisheries Maroochy Research Facility, Nambour, Australia; 5 Diagnostic Laboratory Services, Department of Primary Industries and Regional Development, South Perth, WA, Australia; 6 Biosecurity and Animal Welfare, Department of Industry, Tourism and Trade, Darwin, Australia

**Keywords:** *Mollicutes*, phylogenomics, phytopathogen, ViLL, *Vigna* little leaf phytoplasma

## Abstract

Gene- and genome-based approaches were used to determine whether *Vigna* little leaf (ViLL) phytoplasma, which occurs in northern Australia, is a distinct ‘*Candidatus* Phytoplasma’ species. The ViLL 16S rRNA gene sequences exhibited the highest known similarity to species in the 16SrXXIX-A and 16SrIX-D subgroups, namely ‘*Candidatus* Phytoplasma omanense’ (98.03–98.10%) and ‘*Candidatus* Phytoplasma phoenicium’ (96.87–97.20%), respectively. A total of 48 single-copy orthologue genes were identified to be shared among the two draft ViLL phytoplasma genomes, 30 publicly available phytoplasma genomes, and one *Acholeplasma laidlawii* genome as the outgroup taxon. Phylogenomic assessments using the 48 shared single-copy orthologue genes supported that ViLL and ‘*Ca*. Phytoplasma phoenicium’ were closely related yet distinct species. The 16S rRNA gene sequence analysis and phylogenomic assessment indicate that ViLL phytoplasmas are a distinct taxon. As such, a novel species, ‘*Candidatus* Phytoplasma vignae’, is proposed. Strain BAWM-336 (genome accession number JAUZLI000000000) detected in *Momordica charantia* (bitter melon) serves as the reference strain of this species, with infected plant material deposited in the Victorian Plant Pathology Herbarium (VPRI) as VPRI 44369.

## Introduction

**Fig. 1.**
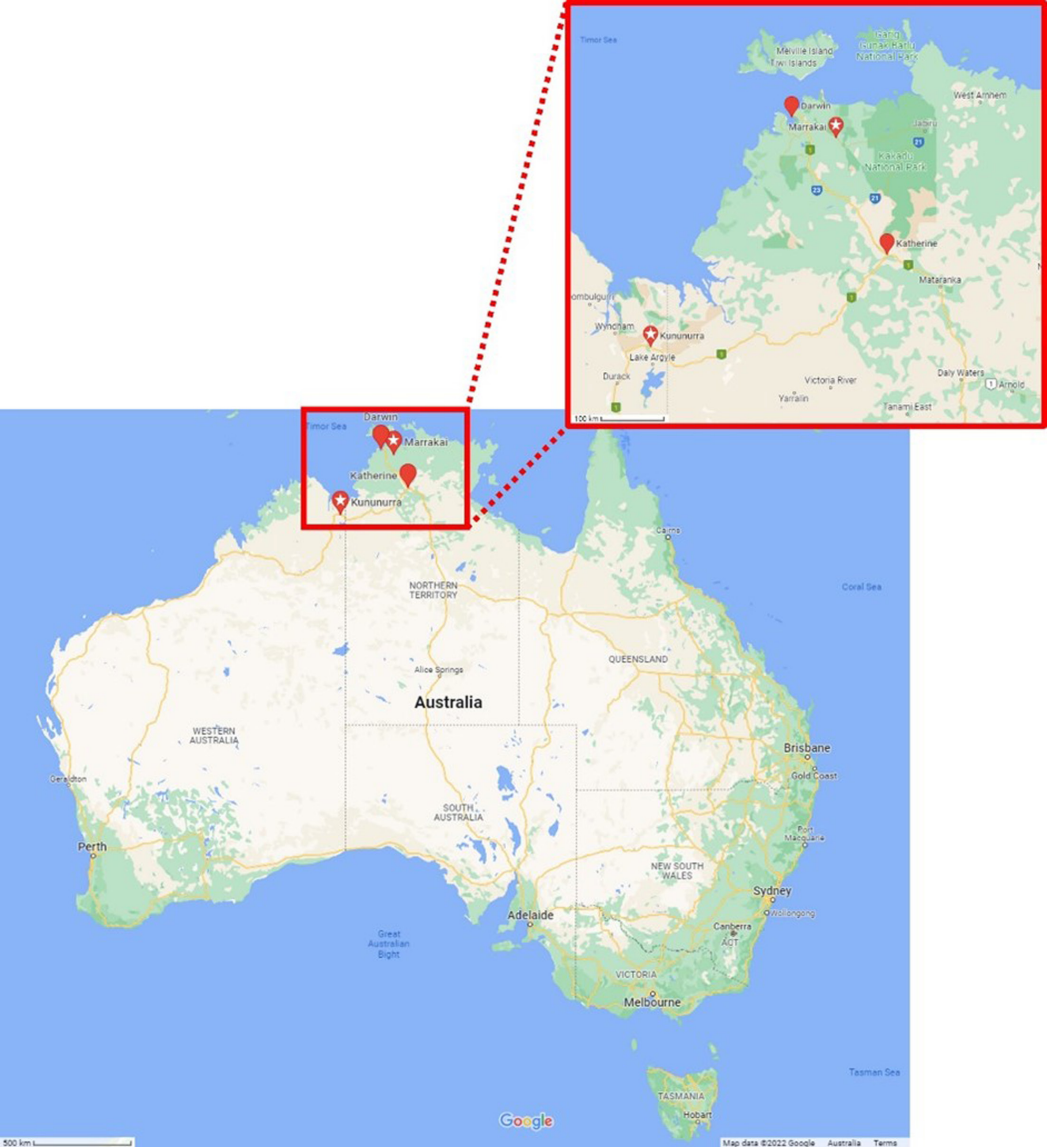
Approximate geographic locations of *Vigna* little leaf detections in Australian surveys. Solid red pins indicate historical detections according to [1,3], while pins with a white centre star indicate the locations of BAWM-245 and BAWM-336 used in this study.

The *Vigna* little leaf (ViLL) phytoplasma has historically been reported in the Northern Territory, Australia, near the townships of Katherine and Darwin (Fig. 1). It was named for the little leaf symptoms associated with its infection of *Vigna lanceolata* (pencil yam, family: *Fabaceae*), first detected in Katherine [1]. Based on Sanger sequencing and/or restriction fragment length polymorphisim (RFLP) analyses, the ViLL phytoplasma was subsequently found associated with phyllody in *Tridax procumbens* (tridax daisy, family: *Asteraceae*) also collected near Katherine [2], and little leaf symptoms in *Stylosanthes scabra* (shrubby stylo, family: *Fabaceae*) from Darwin [3]. In a few instances, mixed infections of both ViLL and *Stylosanthes* little leaf (StLL) phytoplasmas (now described as ‘*Candidatus* Phytoplasma stylosanthis’ [4]) were reported [3,5].

In 2021, ViLL phytoplasmas were detected in a single *Catharanthus roseus* (periwinkle, family *Apocynaceae*) plant showing symptoms of yellowing and little leaves sampled from a private garden in Kununurra, Western Australia [6], and in *Momordica charantia* (bitter melon, family *Cucurbitaceae*) sampled from a single symptomatic plant collected from a field in Marrakai, Northern Territory (Fig. 1). Disease in this bitter melon crop was unusually high, with between 70 and 80% of the *M. charantia* crop showing symptoms of phyllody, distorted fruit shape, and witches’-broom (Samantha Bond, personal communication). These ViLL phytoplasma detections made in 2021 represent a new report for Western Australia and expand the known host range of the phytoplasma into two new plant host families, namely the families *Apocynaceae* and the *Cucurbitaceae*. Based on the recent and historical detections, this phytoplasma may be restricted to the northern regions of Australia and may have a diverse host range. However, the ViLL phytoplasma remains an infrequently detected taxon in field surveys conducted in Australia [1,5, 6].

Phytoplasmas are unculturable microbes and, as such, the phenotypic properties of species cannot be assessed directly. The polyphasic approach of combining phenotypic and genealogical traits in a species description are therefore not possible for this group of bacteria. Instead, genealogical evidence and biological properties, when available, are used to delimit a novel phytoplasma species [7,8]. According to the Phytoplasma Taxonomy Group within the International Research Programme for Comparative Mycoplasmology (IRPCM), a novel ‘*Ca*. Phytoplasma’ taxon can be described when the 16S rRNA gene sequence bears less than 98.65% nucleotide sequence similarity to previously described ‘*Ca*. Phytoplasma’ taxa [8]. In cases where the 16S rRNA gene sequence is more than 98.65% similar to a previously described taxon but has a distinct vector and host range or is associated with distinct symptomology in the same host, and there is evidence of significant molecular or serological diversity, a novel taxon can still be assigned. The use of either multilocus sequence analysis or genome sequence comparisons are recommended and implemented in novel species descriptions [9,11], especially when the 16S rRNA gene sequence similarity is higher than the 98.65% threshold [8].

## Sequence analyses of the ViLL phytoplasmas

Although previous studies indicated that the ViLL phytoplasma is a distinct taxon based on the 16S rRNA gene sequence [1,3,5], it has not been characterized as a novel ‘*Ca*. Phytoplasma’ species and is therefore not considered in phylogenetic analyses. Here, we investigate the phylogenetic relationship of ViLL phytoplasmas detected in symptomatic periwinkle and bitter melon plants using 16S rRNA nucleotide sequence similarity and phylogenetic analyses, as well as a phylogenomic approach (Table 1).

**Table 1.** Sample details of two ViLL phytoplasmas analysed in this study including sample name, host species, symptoms and sampling location in Australia as well as DNA paired-end metagenomic sequence data quality evaluations and genome assembly statistics Data quality evaluations include the percent of raw reads passing filter and total reads per library per sample. Genome assembly statistics include sequence depth coverage, G+C content and genome size of ViLL phytoplasma genomes assembled and analysed in this study for phylogenetic and phylogenomic evaluations.

Details	BAWM-245	BAWM-336
Victorian Plant Pathology Herbarium (VPRI) accession no**.**	VPRI 44368	VPRI 44369
Genome accession number	JAUZJL000000000	JAUZLI000000000
Host common name(species name)	Periwinkle(*Catharanthus roseus*)	Bitter melon(*Momordica charantia*)
Host symptoms	Leaf yellowing, mild leaf size reduction, mild proliferation	Phyllody, distorted fruit shape, witches'-broom
Sampling location	Kununurra, Western Australia	Marrakai, Northern Territory
Total reads before QC (million)	7.0	14.61
Total reads after QC (million)	6.9	14.46
Genome size (bp)	540 232	586 186
Contigs >1000 bp (*n*)	34	28
Coverage (×)	233.0	99.3
N50 (bp)	29 777	76 363
G+C content (mol%)	23.4	24.0
tRNA genes (*n*)	33	32
rRNA genes (*n*)	1	1
Protein-coding genes (*n*)	459	472

Total DNA was extracted from symptomatic petiole and leaf tissue, and the DNA quality and quantity assessed according to [4]. All total DNA extractions were stored at −20 °C until use. Total DNA extracts were used in a nested PCR using P1/P7 primers followed by R16F2n/m23sr primers for 16S rRNA-based phytoplasma identification [12] and sent for Sanger sequencing after size confirmation by agarose gel electrophoresis according to [4]. Each amplicon sequence per sample was assembled into a consensus sequence using Geneious Prime 2022.1.1 (https://www.geneious.com) and submitted for blastn analyses [13] at the NCBI (https://blast.ncbi.nlm.nih.gov/Blast.cgi). Both assembled amplicons showed over 99.7% sequence similarity to the 16S rRNA gene from previously submitted ViLL phytoplasmas (Genbank accession numbers: Y15866 and AJ289195), indicating detections of ViLL phytoplasmas in the periwinkle and bitter melon hosts. The phytoplasma-positive plant material was preserved by freeze drying in individual screw cap tubes for at least 72 h at −50 °C using the FreeZone 2.5 l Benchtop Freeze Dry System (Labconco) and submitted to the Victorian Plant Pathology Herbarium (VPRI).

Illumina high-throughput sequencing libraries were prepared, quantified, and sequenced on the Illumina NovaSeq 6000 platform with the SP Reagent Kit version 1.5 as described previously [14]. FastP [15] was used to discard sequences shorter than 50 bp and to remove adapters and sequences below the Q20 threshold. Approximately 6.9 million reads remained for BAWM-245 (98.59% reads passing filters; Table 1), and 14.46 million reads remained for BAWM-336 (98.91% reads passing filters; Table 1). Trimmed reads were assembled and phytoplasma-derived contigs identified and retrieved according to [14]. Retrieved phytoplasma-derived contigs that were shorter than 1000 bp were removed from the assembly. The remaining phytoplasma-derived contigs were submitted to metaQUAST [16] to estimate the G+C content, genome size and N50 values. The phytoplasma genome assembly from sample BAWM-336 was the largest of the two assemblies at 586 186 bp, with the largest N50 value (76 363 bp) (Table 1). The phytoplasma genome assembly from sample BAWM-245 was smaller at 540 232 bp with an N50 value of 29 777 bp (Table 1). The G+C content was 23.4 and 24.0 mol% for BAWM-245 and BAWM-336, respectively. Each of the phytoplasma genome sequences were also used as the reference in BBSplit 38.61 within the BBMap version 38.61b software suite [17] with the corresponding trimmed reads used as input to estimate the coverage of each genome. The genome sequence of BAWM-245 and BAWM-336 had high coverage at 233.0× and 99.3×, respectively. Prokka 1.14.5 [18] was used to predict and count the number of protein coding genes, tRNA genes, and rRNA genes per genome, specifying RNAmmer for rRNA prediction [19]. For the BAWM-245 draft phytoplasma genome, 33 tRNA genes, one rRNA gene, and 459 protein-coding genes were annotated. For the BAWM-336 draft genome, 32 tRNAs, one rRNA gene, and 472 protein-coding genes were annotated (Table 1). Together, the genome assembly statistics and gene annotations above suggest sufficient data and contiguity of the draft ViLL phytoplasma genome sequences for use in comparative genomic and phylogenomic assessments [11,20].

## Evidence of sufficient molecular divergence to assign a novel ‘*Candidatus* phytoplasma’ taxon

### 16S rRNA gene sequence analysis

The 16S rRNA genes were extracted from the genome assemblies according to length recommendations specified by [8]. For the extraction, the 16S rRNA sequences corresponding to representative ‘*Ca*. Phytoplasma’ taxa were compiled into a reference set of sequences [8]. The reference set was mapped back to the ViLL phytoplasma genome assemblies to extract the gene regions using BBMap [17] implemented in Geneious prime 2022.1.1 (https://www.geneious.com). The extracted gene regions were submitted for blastn analysis [13] to confirm their identities for each sample. The extracted 16S rRNA genes were aligned using mafft [21] with previously described ‘*Ca*. Phytoplasma’ representatives to estimate the sequence similarities and phylogenetic position of the ViLL phytoplasmas. Pairwise sequence similarities between the two ViLL phytoplasma 16S rRNA gene sequences extracted from the genome assemblies as well as the original sequences deposited on the NCBI database shared 99.80–99.87% nucleotide sequence identity with each other (Table 2). The ViLL phytoplasma 16S rRNA gene sequences exhibited the next highest sequence similarity to ‘*Ca*. Phytoplasma omanense’ strain IM-4(98.03 %–98.10 %), ‘*Ca*. Phytoplasma phoenicium’ strain SA213 (97.12%–97.20%), and ‘*Ca*. Phytoplasma phoenicium’ strain A4 (96.87%). However, the 16S rRNA gene sequences of ‘*Ca*. Phytoplasma omanense’ strain IM-4 and ‘*Ca*. Phytoplasma phoenicium’ strain SA213 are 90 and 264 bp shorter than the recommended length, respectively, suggesting that the percent identities might differ should full-length 16S rRNA gene sequences for these strains become available. According to the phytoplasma species description guidelines published in 2022 [8], these results support the description of ViLL phytoplasma as a novel species as they exhibit less than 98.65% sequence similarity to the 16S rRNA gene of any previously described ‘*Ca*. Phytoplasma’ species.

**Table 2.** Pairwise sequence similarity analyses among the *Vigna* little leaf (ViLL) phytoplasma strains BAWM-245 and BAWM-336 as well as strains of their close relatives, namely ‘*Candidatus* Phytoplasma phoenicium’ strain SA213 and ‘*Candidatus* Phytoplasma omanense’ strain IM-4 Sequence similarity analyses of the 16S rRNA gene was done for both ViLL strains and their two close ‘*Candidatus* Phytoplasma’ relatives.

Samples in pairwise comparison	16S rRNA gene sequence similarity (%)
BAWM-336 vs BAWM-245	99.80
BAWM-336 vs ‘*Ca*. Phytoplasma phoenicium’ strain SA213	97.20
BAWM-245 vs ‘*Ca*. Phytoplasma phoenicium’ strain SA213	97.12
BAWM-336 vs ‘*Ca*. Phytoplasma phoenicium’ strain A4	96.87
BAWM-245 vs ‘*Ca*. Phytoplasma phoenicium’ strain A4	96.87
BAWM-336 vs ‘*Ca*. Phytoplasma omanense’ strain IM-4	98.03
BAWM-245 vs ‘*Ca*. Phytoplasma omanense’ strain IM-4	98.10

**Fig. 2.**
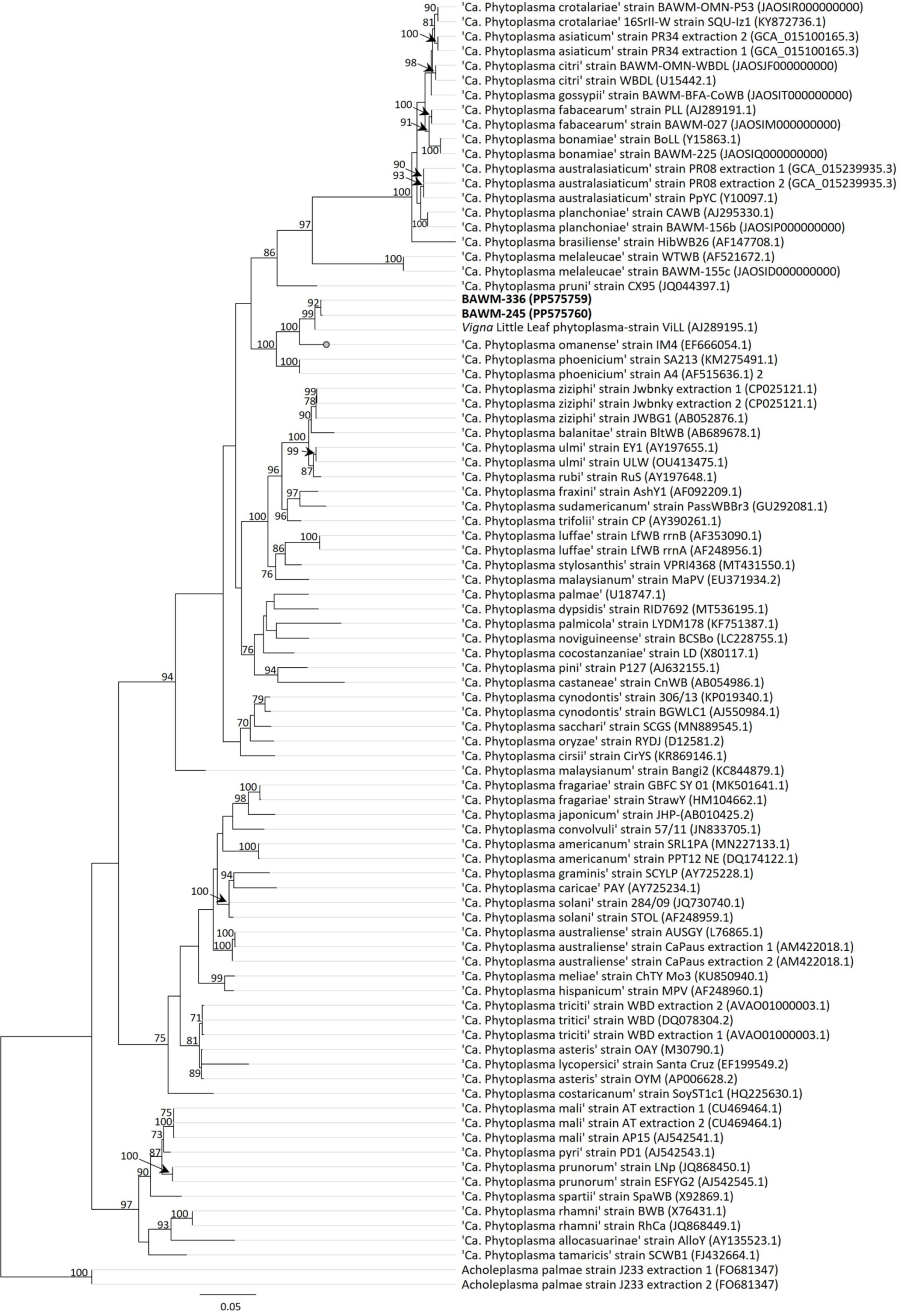
Maximum-likelihood tree inferred from analysis of the 16S rRNA gene sequences of two *Vigna* little leaf phytoplasma strains (highlighted in bold font) and previously described ‘*Candidatus* Phytoplasma’ reference and related strains. *Acholeplasma palmae* was used as the outgroup taxon. Maximum- likelihood analyses were performed using RaxML implemented in Geneious Prime 2022.1.1. Bootstrap support values>70% are indicated at branch nodes, and branch lengths indicate the number of nucleotide substitutions per site (see bar).

Alignments of the 16S rRNA genes were used to reconstruct a maximum-likelihood tree using RAxML version 4 implemented using Geneious Prime 2022.1.1 to infer the phylogenetic position of the ViLL phytoplasma strains (bootstrap method with 1000 bootstrap replicates [22]). The phylogenetic analyses of the 16S rRNA gene indicate that the ViLL phytoplasmas form their own, well-supported clade (bootstrap support of 99%), with a close evolutionary relationship to strains of ‘*Ca*. Phytoplasma omanense’ and ‘*Ca*. Phytoplasma phoenicium’ (Fig. 2).

### Phylogenomic comparisons

Scaffolds of non-phytoplasma origin were identified in the publicly available draft genome sequence of ‘*Ca*. Phytoplasma phoenicium’ strain SA213 (GenBank accession no.: NZ_JPSQ00000000.1) using blastn analyses in previous assessments (data not shown). To ensure accurate comparisons between the genomes analysed in the study, the non-phytoplasma scaffolds were removed from the assembly and used in all further downstream analyses. The removal of the non-phytoplasma scaffolds reduced the ‘*Ca*. Phytoplasma phoenicium’ genome size from 345 965 to 312  035 bp and reduced the number of protein coding genes from 314 to 275. The ‘*Ca*. Phytoplasma phoenicium’ genome was further determined to be highly incomplete due to the low number of tRNA genes recovered from the genome (three tRNA genes in both the contaminated and contaminated-removed genomes).

Phylogenomic comparisons between the two ViLL genomes, 30 publicly available phytoplasma genomes, and one *Acholeplasma laidlawii* genome (Table S1, available in the online version of this article) were performed as previously described [11]. Briefly, this approach implements Orthofinder 2 [23,24] to identify the single-copy orthologue genes shared between all genomes considered. A total of 48 shared single-copy orthologue genes were identified among the 33 genomes used in the analyses. The function of the single copy orthologue genes obtained by Prokka annotation are listed in Table S2. The 48 shared single-copy orthologue genes were individually aligned using mafft [21], and then analysed in iq-tree version 2.0.5 [25,27] to concatenate and construct the subsequent phylogenomic tree. Branch support was shown using gene concordance factor (gCF) and site concordance factor (sCF) values, with gCF and sCF values of ≥50% indicating high statistical support.

**Fig. 3.**
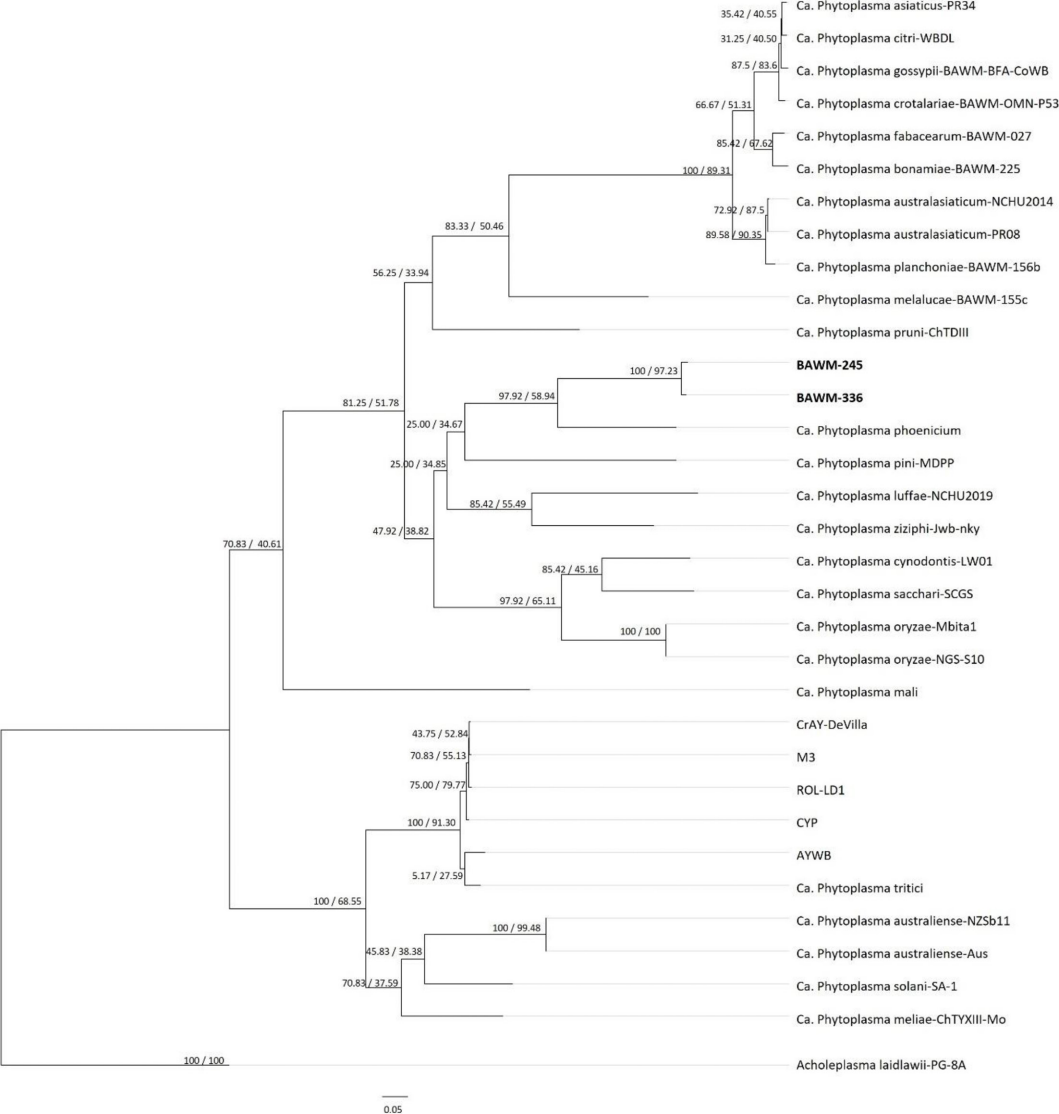
Maximum-likelihood tree inferred using iq-tree version 2.0.5 using the concatenated dataset of 48 single copy orthologue genes identified to be shared across each of the 33 genomes analysed in this study. Support values include gene (gCF) and site concordance factors (sCF), i.e., gCF / sCF. Branch lengths indicate the number of amino acid substitutions per site (see bar). Bold font indicates the *Vigna* little leaf (ViLL) phytoplasma strains analysed in this study.

The high gCF (100%) and sCF (97.23%) values of the ViLL branch in the phylogenomic tree and their short respective branch lengths support that the two strains are closely related. Additionally, the branch encompassing the two ViLL phytoplasma strains and ‘*Ca*. Phytoplasma phoenicium’ strain SA213 show high gCF (97.92%) and sCF (58.94%) support values supporting that the ViLL strains are distinct species from ‘*Ca*. Phytoplasma phoenicium’ (Fig. 3). Unfortunately, no genome sequences were available for comparison with the known ViLL relative, ‘*Ca*. Phytoplasma omanense’ at the time of this study. Additionally, the genome of ‘*Ca*. Phytoplasma phoenicium’ strain SA213 is highly incomplete and therefore limited the number of single-copy orthologues obtained for the phylogenomic analyses (Table S2).

## Description of ‘*Candidatus* phytoplasma vignae’

‘*Candidatus* Phytoplasma vignae’ (vig’nae N.L. gen. n. *vignae*, of *Vigna*, referring to the plant genus in which the phytoplasma was first detected).

The reference strain is BAWM-336^R^ associated with *Momordica charantia* (bitter melon) showing symptoms of phyllody, fruit shape distortions, and witches'-broom. [Mollicutes] NC; NA; O, wall-less; NAS (GenBank accession number: JAUZLI000000000); G+C content 24.0 mol%; oligonucleotide sequences of unique regions of the 16S rRNA gene are shown in Table 3.

**Table 3.** Unique oligonucleotide sequences and their position in the 16S rRNA gene of ‘*Ca*. Phytoplasma vignae’ reference strain BAWM-336^R^

Position	Unique sequence (5′ to 3′ orientation)
61–137 bp	ACCACGAAAGTTGGCAATACCCAAAAGCGGTCGCCTAACTTCTTCGGAAGAGGGAGCCGTCTAAGGTAGGGTCGATG
301–306 bp	ACTGTG
358–453 bp	CCTAAAACGAGCGCAACCCTTATCGTTAGTTGCGACCACGTAATGGTGAGCACTTTAGCGAGACTGCCAATGATAAATTGGAGGAAGGTGAGGATT
507–583 bp	GAAGATACACGAAAAACCTTACCAGGTCTTGACATAATTTTGCGAAGTTATAGAAATATAACGGAGGTTATCAGAAT
569–633 bp	TTTGGTAAGTCTATAGTTTAATTTCAGCGCTTAACGCTGTTGCGCTATAGAAACTGCCTCACTA
1271–1323 bp	AAGGTATACTTAAAGAGGGGCTTGCGTCACATTAGTTAGTTGGTAAGGTAATG

Preserved *M. charantia* (bitter melon) tissue of sample BAWM-336^R^ from Marrakai, Northern Territory, Australia, was deposited at the Victorian Plant Pathology Herbarium (VPRI) under the accession VPRI 44369 at Agriculture Victoria, Bundoora, Australia. Sample BAWM-245 was also submitted to the VPRI (accession VPRI 44368). We propose that the genome sequence of strain BAWM-336^R^ (GenBank assembly number: JAUZLI000000000) be considered as a reference genome for ‘*Ca*. Phytoplasma vignae’. This strain was selected as the reference strain because it originated from the Northern Territory where ViLL phytoplasmas were first reported [1]. The 16S rRNA nucleotide sequence analyses also indicate a close relationship of strain BAWM-336^R^ with that of previous detections in the Northern Territory (Fig. 2).

The results presented here provide multiple lines of evidence using more than one sample to support the description of ViLL as a novel phytoplasma species, including comparisons of gene sequence identities (16S rRNA gene) and reconstructing phylogenies (16S rRNA gene and shared single-copy orthologues). These analyses are in-line with the updated recommendations for phytoplasma species descriptions [8] and are an improvement over the traditional approaches that only considered the 16S rRNA gene sequence similarity and RFLP [7], which represent a very small fraction of the genome and are much less informative for establishing taxonomic relationships among ‘*Ca*. Phytoplasma’ species.

The genomes obtained in this study serve as an essential resource to aid in improving our understanding of phytoplasma evolution, supporting the epidemiological studies of this species, and advancing phytoplasma diagnostics going forward. The preserved material deposited at the VPRI through this study serves as an important resource that should be revisited to improve the quality and completeness of the genome assemblies of these strains in the future. Putative vectors of ViLL were determined to be *Austroagallia torrida* (Evans) and a *Batracomorphus species* based on direct PCR analyses only, with transmission by these vectors not yet tested [2]. More work is needed to understand the vector, host, and geographic range of ‘*Ca*. Phytoplasma vignae’. Further work to generate high quality genome sequence data from the close known relatives ‘*Ca*. Phytoplasma phoenicium’ and ‘*Ca*. Phytoplasma omanense’ will improve the understanding of their taxonomic relationship to ‘*Ca*. Phytoplasma vignae’.

## supplementary material

10.1099/ijsem.0.006502 Uncited Table S1.

## Abbreviations

gCF: gene concordance factor
IRPCM: International Research Programme on Comparative Mycoplasmology
RFLP: restriction fragment length polymorphisim
sCF: site concordance factor
StLL: stylosanthes little leaf
ViLL: vigna little leaf
