# Supplementary material for: 'Candidatus Phytoplasma vignae’, assigning a species description to a long-known phytoplasma occurring in northern Australia
Source: Int J Syst Evol Microbiol. 2024 Aug 27;74(8):006502. doi: 10.1099/ijsem.0.006502 (PMC11349051; doi:10.1099/ijsem.0.006502)
Supplement: Uncited Table S1. [file ijsem-74-06502-s001.pdf]

ViLL – Supplementary Tables

Supplementary Table S1: List of publicly available genomes and their accession numbers used for analyses in this study.

**Strain name**	**Accession no.**
‘*Ca.* P. asiaticum’ strain PR34	GCA_015100165.3
‘*Ca.* P. citri’ strain WBDL	JAOSJF000000000
‘*Ca.* P. gossypii’ strain BAWM-BFA-CoWG	JAOSIT000000000
‘*Ca.* P. crotalariae’ strain BAWM-OMN-P53	JAOSIR000000000
‘*Ca.* P. fabacearum’ strain BAWM-027	JAOSIM000000000
‘*Ca.* P. bonamiae’ strain BAWM 225	JAOSIQ000000000
‘*Ca.* P. australasiaticum’ strain PR08	GCA_015239935.3
‘*Ca.* P. planchoniae’ strain BAWM-156b	JAOSIP000000000
‘*Ca.* P. melaleucae’ strain BAWM-155c	JAOSID000000000
‘*Ca.* P. pruni’ strain ChTDIII	GCA_013391955.1
‘*Ca.* P. phoenicium’ strain SA213	GCA_001189415.1
‘*Ca.* P. pini’ strain MDPP	GCA_007821455.1
‘*Ca.* P. luffae’ strain NCHU2019	GCA_001307505.2
‘*Ca.* P. ziziphi’ strain Jwb-nky	GCA_003640545.1
‘*Ca.* P. cynodontis’ strain LW01	GCA_009268075.1
‘*Ca.* P. sacchari’ strain SCGS	GCA_009268105.1
‘*Ca.* P. oryzae’ strain Mbita1	GCA_001578535.1
‘*Ca.* P. oryzae’ strain NGS-S10	GCA_003263355.1
‘*Ca.* P. mali’ strain AT	CU469464.1
‘*Ca.* P. asteris’-related strain CrAY-DeVilla	GCA_004214875.1
‘*Ca.* P. asteris’ strain M3	GCA_001712875.1
‘*Ca.* P. asteris’-related strain ROL-LD1	GCA_001866375.1
‘*Ca.* P. asteris’-related strain CYP	GCA_000803325.1
‘*Ca.* P. asteris’-related strain AYWB	GCA_000012225
‘*Ca.* P. tritici’ strain WBD	NZ_AVAO01000003
‘*Ca.* P. australiense’ strain NZSb11	GCA_000397185.1
‘*Ca.* P. australiense’ strain Aus	GCA_000069925.1
‘*Ca.* P. solani’ strain SA-1	GCF_003698095.1
‘*Ca.* P. meliae’ strain ChTYXIII-Mo	GCA_016876135.2
Acholeplasma laidlawii PG-8A	GCA_000018785.1

Supplementary Table S2: Functional annotations and corresponding locus tags of BAWM-245 an BAWM-336 for the 48 single copy orthologs used in the phylogenomic analyses of 33 phytoplasma genomes. The functional annotations were provided during the Prokka analyses while the orthogroup names are assigned by Orthofinder 2.

No.	Orthogroup name	Functional annotation	Locus tag (BAWM-245)	Locus tag (BAWM-336)
1	OG00126	Hypothetical protein	Q8784_01935	Q8888_02275
2	OG00127	Phosphatidylserine decarboxylase proenzyme 2	Q8784_01895	Q8888_01610
3	OG00128	CTP synthase	Q8784_00645	Q8888_00300
4	OG00129	Hypothetical protein	Q8784_00650	Q8888_00295
5	OG00130	Hypothetical protein	Q8784_00655	Q8888_00290
6	OG00131	Hypothetical protein	Q8784_00660	Q8888_00285
7	OG00133	DNA polymerase III subunit alpha	Q8784_00700	Q8888_00245
8	OG00134	Asparagine synthetase B [glutamine-hydrolyzing]	Q8784_00680	Q8888_00265
9	OG00135	Hypothetical protein	Q8784_00685	Q8888_00260
10	OG00136	Phenylalanine--tRNA ligase alpha subunit	Q8784_02435	Q8888_00735
11	OG00137	Phenylalanine--tRNA ligase beta subunit	Q8784_02440	Q8888_00740
12	OG00138	Vitamin B12 import ATP-binding protein BtuD	Q8784_02025	Q8888_00750
13	OG00139	Pyruvate dehydrogenase E1 component subunit alpha	Q8784_02030	Q8888_00755
14	OG00140	Pyruvate dehydrogenase E1 component subunit beta	Q8784_02035	Q8888_00760
15	OG00141	Dihydrolipoyllysine-residue acetyltransferase component of pyruvate dehydrogenase	Q8784_02040	Q8888_00765
16	OG00142	Dihydrolipoyl dehydrogenase	Q8784_00910	Q8888_01015
17	OG00143	Hypothetical protein	Q8784_01265	Q8888_00625
18	OG00152	Hypothetical protein	Q8784_00900	Q8888_01005
19	OG00153	Hypothetical protein	Q8784_00895	Q8888_01000
20	OG00154	Single-stranded DNA-binding protein	Q8784_00890	Q8888_00995
21	OG00155	Hypothetical protein	Q8784_02115	Q8888_00840
22	OG00156	Hypothetical protein	Q8784_02120	Q8888_00845
23	OG00158	Hypothetical protein	Q8784_01970	Q8888_00180
24	OG00159	Hypothetical protein	Q8784_01965	Q8888_00185
25	OG00160	Hypothetical protein	Q8784_01710	Q8888_00085

26	OG00161	Hypothetical protein	Q8784_01775	Q8888_01810
27	OG00162	Hypothetical protein	Q8784_02050	Q8888_00775
28	OG00163	Hypothetical protein	Q8784_02055	Q8888_00780
29	OG00169	Hypothetical protein	Q8784_00210	Q8888_01230
30	OG00172	Hypothetical protein	Q8784_01550	Q8888_01855
31	OG00173	Hypothetical protein	Q8784_00580	Q8888_00365
32	OG00174	Hypothetical protein	Q8784_00575	Q8888_00370
33	OG00175	Hypothetical protein	Q8784_00365	Q8888_01055
34	OG00177	Hypothetical protein	Q8784_00500	Q8888_00445
35	OG00178	Hypothetical protein	Q8784_00495	Q8888_00450
36	OG00182	DNA primase	Q8784_00505	Q8888_00440
37	OG00185	Hypothetical protein	Q8784_00025	Q8888_01420
38	OG00187	Hypothetical protein	Q8784_00835	Q8888_00940
39	OG00188	Hypothetical protein	Q8784_01790	Q8888_01825
40	OG00190	Hypothetical protein	Q8784_02070	Q8888_00795
41	OG00191	Hypothetical protein	Q8784_02080	Q8888_00805
42	OG00192	ATP-dependent zinc metalloprotease FtsH	Q8784_02085	Q8888_00810
43	OG00193	Hypothetical protein	Q8784_01995	Q8888_00155
44	OG00194	Hypothetical protein	Q8784_01870	Q8888_01585
45	OG00195	Hypothetical protein	Q8784_01590	Q8888_01960
46	OG00204	Hypothetical protein	Q8784_00725	Q8888_00220
47	OG00205	Hypothetical protein	Q8784_00720	Q8888_00225
48	OG00206	Hypothetical protein	Q8784_00710	Q8888_00235
